# Progranulin promotes the retinal precursor cell proliferation and the photoreceptor differentiation in the mouse retina

**DOI:** 10.1038/srep23811

**Published:** 2016-03-31

**Authors:** Yoshiki Kuse, Kazuhiro Tsuruma, Sou Sugitani, Hiroshi Izawa, Yuta Ohno, Masamitsu Shimazawa, Hideaki Hara

**Affiliations:** 1 Molecular Pharmacology, Department of Biofunctional Evaluation, Gifu Pharmaceutical University, 1-25-4 Daigaku-nishi, Gifu 501-1196, Japan

## Abstract

Progranulin (PGRN) is a secreted growth factor associated with embryo development, tissue repair, and inflammation. In a previous study, we showed that adipose-derived stem cell-conditioned medium (ASC-CM) is rich in PGRN. In the present study, we investigated whether PGRN is associated with retinal regeneration in the mammalian retina. We evaluated the effect of ASC-CM using the *N*-methyl-*N*-nitrosourea-induced retinal damage model in mice. ASC-CM promoted the differentiation of photoreceptor cells following retinal damage. PGRN increased the number of BrdU^+^ cells in the outer nuclear layer following retinal damage some of which were Rx (retinal precursor cell marker) positive. PGRN also increased the number of rhodopsin^+^ photoreceptor cells in primary retinal cell cultures. SU11274, a hepatocyte growth factor (HGF) receptor inhibitor, attenuated the increase. These findings suggest that PGRN may affect the differentiation of retinal precursor cells to photoreceptor cells through the HGF receptor signaling pathway.

Retinal photoreceptor cell loss can severely impair vision. Dry age-related macular degeneration (AMD) and retinal pigmentosa (RP) are two conditions associated with photoreceptor degeneration^1^,^2^, for which there are currently no effective drug therapies. A recent preclinical study reported the transplantation of induced pluripotent stem (iPS) cell-derived retinal pigment epithelium (RPE) cell sheets into the subretinal space of monkeys^3^. A clinical trial is currently underway to study the clinical application of iPS cells for the treatment of retina disease in humans. However, this clinical trial is focused on the treatment of wet not dry AMD and the therapy cannot regenerate damaged photoreceptor cells.

Progranulin (PGRN) is a secreted growth factor associated with embryonic development^4^, tissue repair^5^, and inflammation^6^,^7^. PGRN expression is mainly observed in the brain in neurons and microglia^8^. PGRN gene mutations cause the accumulation of TAR DNA-binding protein 43 (TDP-43) and have been identified as a cause of frontotemporal lobar degeneration and amyotrophic lateral sclerosis (ALS)^8^,^9^. PGRN can modulate the Wnt signaling pathway^10^, which is associated with cell proliferation and development^11^,^12^. Wnt3a has been shown to increase neurogenesis in the hippocampus^13^ and also to promote retinal regeneration in rats and mice^14^. We have previously identified PGRN as a major component of mouse adipose-derived stem cell (ASC)-conditioned medium (ASC-CM)^15^. It was found to inhibit light-induced retinal photoreceptor cell damage both *in vitro* and *in vivo*. Moreover, the injection of ASC after retinal damage attenuated the decrease in retinal function and thickness without engraftment. These results suggest that some ASC-secreted factors may promote the regeneration of retinal cells.

It has long been thought that mammalian neurons are incapable of regenerating after being damaged. However, recently some reports have suggested that neuronal regeneration can occur in regions of the central nervous system such as the brain and retina in adult mammals^14^,^16^,^17^,^18^,^19^. Müller glial cells have the potential as retinal stem cells to proliferate and dedifferentiate to retinal progenitor cells after retinal injury^14^,^19^. Subsequently, some of these retinal progenitor cells migrate to other any retinal cell layers and differentiate into retinal cells^19^,^20^. In the present study, we showed ASC-CM and PGRN can be contributed to the retinal precursor cell proliferation and photoreceptor differentiation. PGRN can have the potential to promote the retinal regeneration during retinal degeneration.

## Results

### ASC-CM increased rhodopsin^+^ BrdU^+^ cells after retinal damage

**Figure 1:**
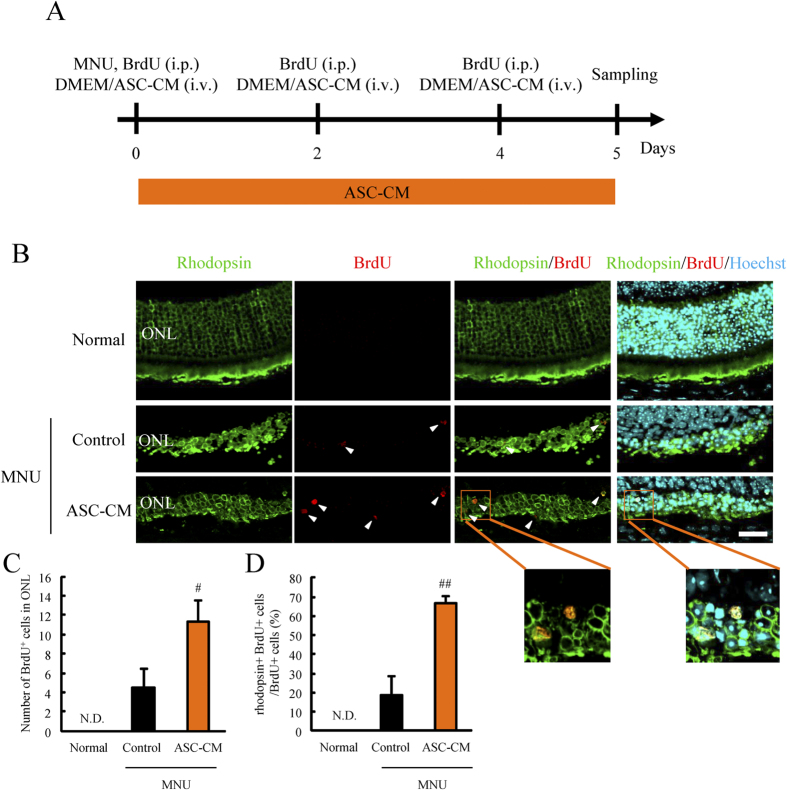
ASC-CM promoted retinal photoreceptor differentiation following MNU-induced retinal damage. (**A**) Experimental procedure. Mice were injected MNU and BrdU by intraperitoneal (i.p.) injection. Following this the mice were treated with 150-fold concentrated vehicle (DMEM) or ASC-CM by intravitreal (i.v.) injection. At 2 and 4 days post MNU treatment, mice were similarly treated with vehicle or ASC-CM. After 5 days, mice eyes were enucleated. (**B–D**) Typical immunostaining images showing rhodopsin (green) and BrdU (red) staining. BrdU^+^ cell were not observed in the ONL in the normal group. Some BrdU^+^ cells were observed in the control group and ASC-CM increased the number of BrdU^+^ cells in the ONL. ASC-CM also increased the number of rhodopsin^+^ BrdU^+^ cells. An enlarged image shows the presence of rhodopsin^+^ BrdU^+^ cells in the ONL. Data are shown as means ± S.E.M. (n = 6). ^##^p < 0.01 and ^#^p < 0.05 vs. control (Student’s *t*-test). Scale bar = 20 μm.

We first examined the effects of ASC-CM on retinal regeneration after retinal damage. ASC-CM treatment after retinal damage increased the number of BrdU^+^ cells in the outer nuclear layer (ONL) compared to a vehicle-treated (control) group and no BrdU^+^ cells were observed in a normal group (Fig. 1B,C). ASC-CM also increased BrdU^+^ cells in ganglion cell layer (GCL) and retinal nerve fiber layer (RNFL) compared to control group (Supplementary Fig. S1B). BrdU^+^ cells were colocalized with GFAP^+^ cells, but not Brn3a^+^ cells in RNFL (Supplementary Fig. S1C). Most significantly, ASC-CM increased rhodopsin^+^ BrdU^+^ cells compared to the control group (Fig. 1B,D). These results suggest that ASC-CM promotes the differentiation of retinal progenitor cells to photoreceptor cells.

### PGRN treatment after retinal damage increased the number of BrdU^+^ cells in the ONL

**Figure 2:**
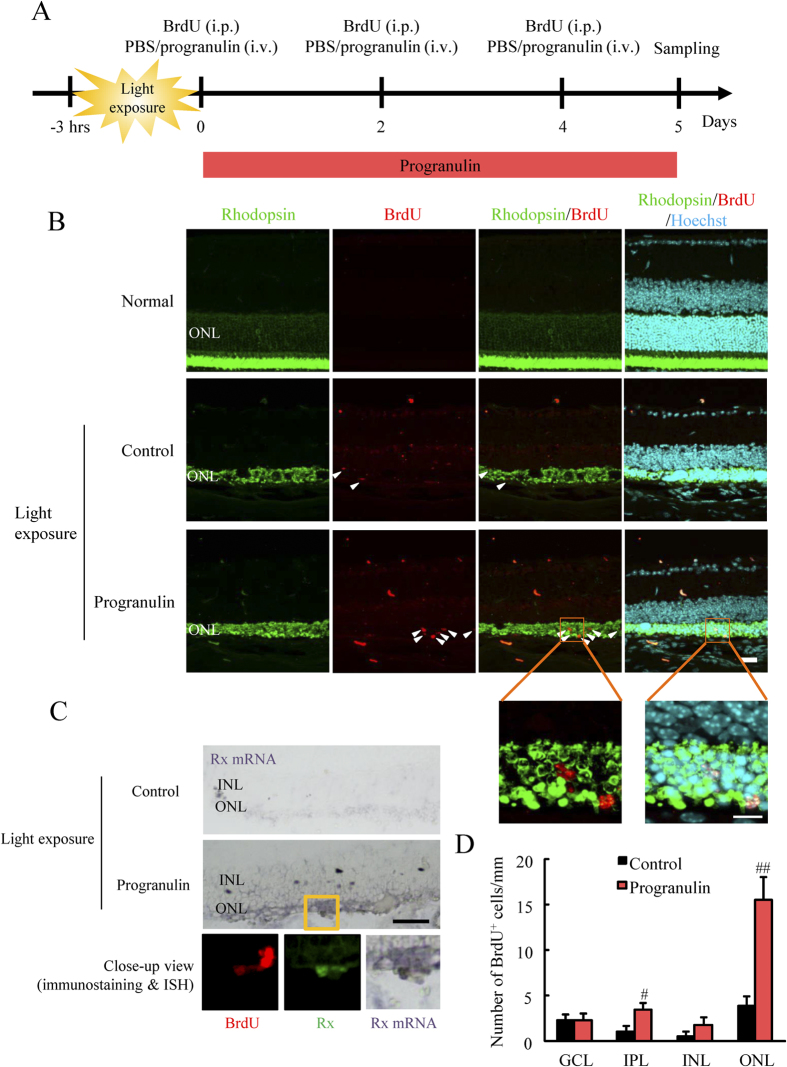
PGRN increased BrdU^+^ cell numbers following light-induced retinal damage. (**A**) Experimental procedure. Mice were exposed to visible light (8,000 lux) for 3 h. After light exposure, mice were administered BrdU by intraperitoneal (i.p.) injection and mice were treated with vehicle (PBS) or PGRN 2 μL by intravitreal (i.v.) injection. At 2 and 4 days post light-induced retinal damage, mice were similarly treated with vehicle or PGRN. After 5 days, mice eyes were enucleated. (**B,D**) Typical images of immunostaining showing rhodopsin (green) and BrdU (red). BrdU^+^ cells were not observed in the normal group. Some BrdU^+^ cells were observed in the control group and PGRN increased the number of BrdU^+^ cells specifically in the ONL. Close-up images indicate BrdU^+^ cells were rhodopsin negative. (**C**) The localization of *Rx* mRNA by *in situ* hybridization (ISH). *Rx* mRNA was colocalized with Rx protein and BrdU in PGRN-treated ONL but not control group as shown in close-up view. Data are shown as means ± S.E.M. (n = 9). ^##^p < 0.01 and ^#^p < 0.05 vs. control (Student’s *t*-test). Scale bar = 20 μm, 10 μm.

In a previous reported we found that ASC-CM contained a high concentration of PGRN (75 fold ASC-CM equals PGRN 574.15 ng/mL)^15^. To confirm whether the differentiation effect of ASC-CM (Fig. 1) resulted from PGRN using light-induced retinal damage model, a better model similar to a pathology of retinal degenerative diseases, we investigated whether PGRN promotes the differentiation of retinal precursor cells to retinal photoreceptor cells after retinal damage *in vivo*. Recent reports have showed that PGRN is associated with muscle regeneration through the regulation of myogenic progenitor cells^21^. Therefore, we investigated the effect of PGRN on retinal regeneration. No BrdU^+^ cells were observed in any retinal layer in an non-injured normal group (Fig. 2B). PGRN treatment after retinal damage increased the number of BrdU^+^ cells in the inner plexiform layer (IPL) and ONL (approximately 4 fold) compared to the control group (Fig. 2B,D). PGRN had no effect on the number of BrdU^+^ cells in GCL and inner nuclear layer (INL). The BrdU^+^ cells in the PGRN group were not rhodopsin positive in spite of their presence in the ONL (Fig. 2B,D).

### PGRN promoted Rx^+^ retinal precursor cell genesis in the ONL

We observed that PGRN increased the number of BrdU^+^ cells in the ONL. To eliminate the possibility that these BrdU^+^ cells were produced by glial cell proliferation during gliosis, we used double staining with BrdU and glial cell specific markers. No co-staining in the ONL was observed using antibodies to glial fibrillary acidic protein (GFAP), a marker specific for astrocytes, and BrdU in the control or PGRN-treated groups (Supplementary Fig. 2A), with GFAP expression mainly observed in retinal inner layer. Moreover, no co-staining was apparent using antibodies to ionized calcium binding adaptor molecule 1 (Iba-1), a marker specific to microglia and BrdU staining in the ONL in the control or PGRN-treated groups (Supplementary Fig. 2B) with Iba-1 expression mainly observed in the IPL. This data shows that BrdU^+^ cells are not glial cells in the ONL. We also performed immunostaining using retinal precursor cell markers. Pax6 and Rx (retinal homeobox protein) were selected as appropriate retinal precursor cell markers. Pax6 is a transcription factor which is closely associated with eye development^22^, it is expressed in ganglion, amacrine, and retinal precursor cells^23^. Rx is also associated with retinal development and is expressed in retinal precursor cells^24^,^25^. Pax6 labeling in the INL was mainly observed in INL because the amacrine cells were labeled (Supplementary Fig. 2C). Pax6 was not expressed in any cells in the ONL. On the other hand, some Rx^+^ cells were observed in PGRN-treated ONL (Supplementary Fig. 2C). No Rx^+^ cells were observed in the control (vehicle-treated group) group but co-staining of Rx and BrdU showed that a few BrdU^+^ cells in the ONL were Rx^+^ in the PGRN-treated group (Supplementary Fig. 2C,D). However, the high background (non-specific signals) was observed in control and PGRN-treated group. Then, we investigated about the localization of *Rx* mRNA by *in situ* hybridization (ISH). The staining by ISH and immunofluorescense revealed *Rx* mRNA was colocalized with Rx protein and BrdU in PGRN-treated ONL, but not control group (Fig. 2C). This suggested that the increase in BrdU^+^ cells in the ONL resulting from PGRN treatment were a few of Rx^+^ retinal precursor cells. Nestin is a marker of neural progenitors. It is reported that nestin is expressed when the injury induces Müller glial neural stem cell-like properties^14^. Nestin expression in PGRN-treated group was not altered compared to the control group (Supplementary Fig. S3A). Sox2 is a stem cell marker and we observed a few of BrdU and Sox2 double-positive cells in PGRN-treated group (Supplementary Fig. S3B). Moreover, cone-rod homeobox protein (CRX) indicates the presence of retinal photoreceptor precursor cells^29^, and we investigated whether PGRN increased the CRX expression. Light damage did not generate the expression of CRX as seen the control group. CRX expression was observed in the PGRN-treated group (Supplementary Fig. S4). These results suggest that PGRN increased the newly-generated retinal precursor cells in ONL.

### PGRN increased rhodopsin^+^ cells in primary retinal cell culture

**Figure 3:**
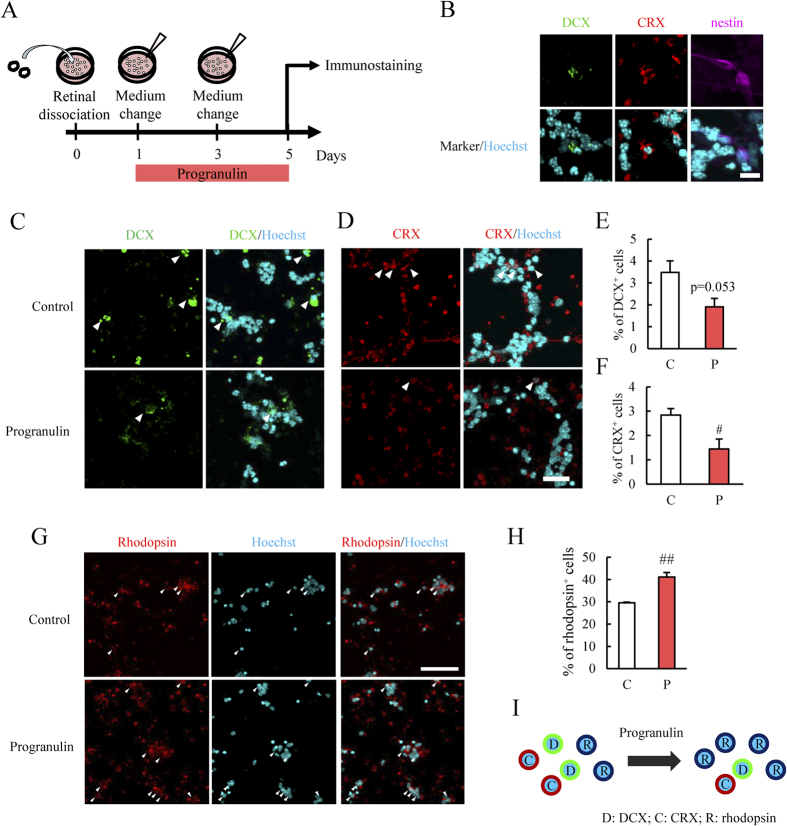
The effect of PGRN on retinal precursor cells in primary culture. (**A**) The eyes from 8-day old mice were enucleated and the retinas were dissected. After dissection the retinas were centrifuged with any reagents. The retinal cells were incubated for 20 h after dissociation. After incubation, the medium was changed and vehicle or PGRN (500 ng/mL) was added to the retinal cell culture. After 3 days, reagents were added to the culture. The cells were collected for western blotting (after 4 days) and for immunostaining (after 5 days). (**B**) The presence of precursor cells in the primary retinal cell culture was confirmed by immunostaining for DCX (neural precursor cells), CRX (photoreceptor precursor cells) and nestin (neural precursor cells). The images show DCX (green), CRX (red), nestin (magenta) and Hoechst 33342 (cyan) staining. (**C–F**) PGRN decreased the number of DCX^+^ cells and CRX^+^ cells compared to controls. Data are shown as means ± S.E.M. (n = 4). ^#^p < 0.05 vs. control (Student’s *t*-test). (**G,H**) Typical immunostaining images showing rhodopsin (red) and Hoechst 33342 (cyan). PGRN treatment increased the number of rhodopsin^+^ cells compared to control. Data are shown as means ± S.E.M. (n = 3 or 4). ^##^p < 0.01 vs. control (Student’s *t*-test). (**I**) PGRN increased the number of rhodopsin^+^ photoreceptor cells and resulted in a decrease in the number of CRX^+^ photoreceptor precursor cells and DCX^+^ neural precursor cells. C: Control; P: PGRN.

To investigate the effect of PGRN in detail, we conducted an experiment with primary retinal cell cultures. Mouse retinas were enucleated at postnatal day 8 (P8). The P8 retina contains immature retinal cells^26^. We investigated whether PGRN can promote the differentiation of retinal precursor cells to photoreceptor cells in primary retinal cell culture. We confirmed the no change in the cell number between control and PGRN-treated group (Supplementary Fig. S5A) to exclude the possibility of the just protective effect by PGRN. We observed the presence of the retinal stem cell marker in primary retinal cell culture (Fig. 3B). Staining of doublecortin (DCX) and nestin indicates the presence of immature neurons^27^,^28^. PGRN decreased the number of retinal precursor cells in primary retinal cell culture (Fig. 3C–F). Importantly, also PGRN increased the number of rhodopsin^+^ cells compared to the control group (Fig. 3G–I).

### SU11274, an HGFR inhibitor, attenuated the promotion of photoreceptor differentiation by PGRN

**Figure 4:**
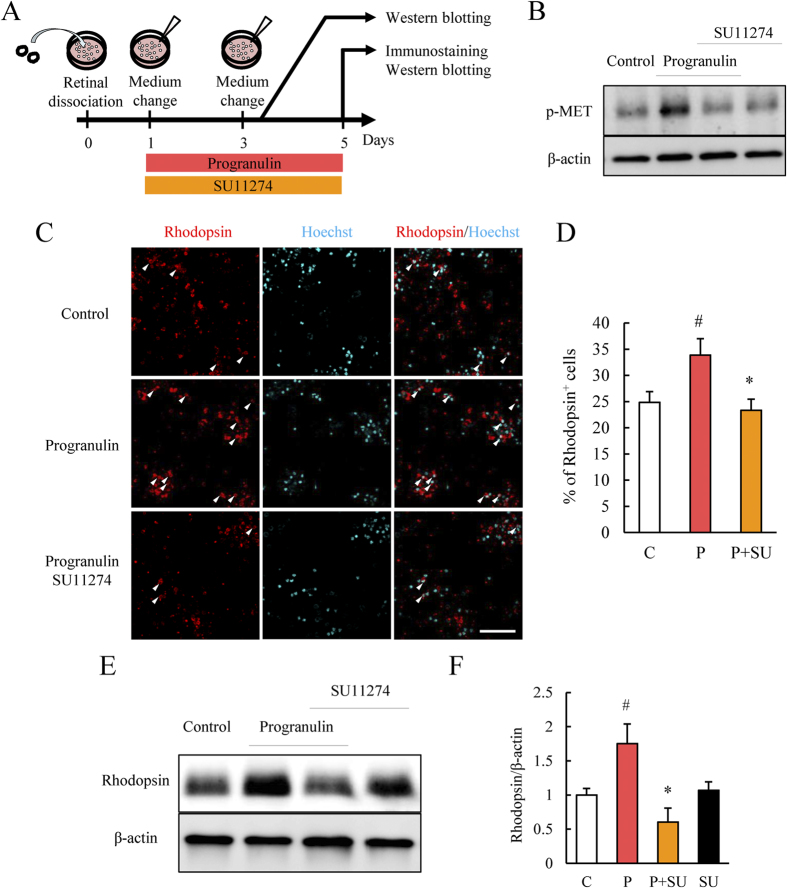
SU11274 treatment inhibited the promotion of differentiation of photoreceptor cells by PGRN. (**A**) After dissociation and incubation, the medium was changed and SU11274 (an HGFR inhibitor) 1 μM and vehicle and PGRN (500 ng/mL) were added in retinal cell culture. After 3 days, the medium was changed maintaining the same composition of additives. The cells were collected for western blotting and for immunostaining after 5 days. (**B**) Immunoblots showing that PGRN increased the phosphorylation level of HGFR following a 5 min incubation. SU11274 prevented the PGRN-induced increase in phosphorylation. (**C,D**) Typical immunostaining images showing rhodopsin (red) and Hoechst 33342 (cyan) staining. PGRN increased the number of rhodopsin^+^ cells compared to control. This effect was blocked by the addition of SU11274. Data are shown as means ± S.E.M. (n = 6 or 7). ^#^p < 0.05 vs. control, *p < 0.05 vs. PGRN (Student’s *t*-test). (**E,F**) Typical blots and quantitative data demonstrating the increase of rhodopsin expression associated with PGRN treatment and the suppression of the increase by SU11274. Treatment with SU11274 alone had no effect on rhodopsin expression level. Data are shown as means ± S.E.M. (n = 3 or 4). ^#^p < 0.05 vs. control, *p < 0.05 vs. PGRN (Student’s *t*-test). C: Control; P: PGRN; SU: SU11274.

We investigated whether HGFR signaling could affect the differentiation induced by PGRN. Immunoblots showed that PGRN treatment increased the phosphorylation of HGFR after 5 min. Co-incubation with the HGFR inhibitor, SU11274, attenuated the phosphorylation by PGRN (Fig. 4B). Next, we confirmed the no change in the cell number in control group, PGRN-treated group and PGRN and SU11274 co-treated group (Supplementary Fig. S5B). Immunostaining results showed that SU11274 at a concentration of 1 μM inhibited the increase of rhodopsin^+^ cells by PGRN (Fig. 4C,D). The increase in rhodopsin expression by PGRN was attenuated by SU11274 treatment (Fig. 4E,F). The addition of SU11274 alone had no effect on rhodopsin expression (Fig. 4E,F).

### PGRN loss caused retinal neuron loss

**Figure 5:**
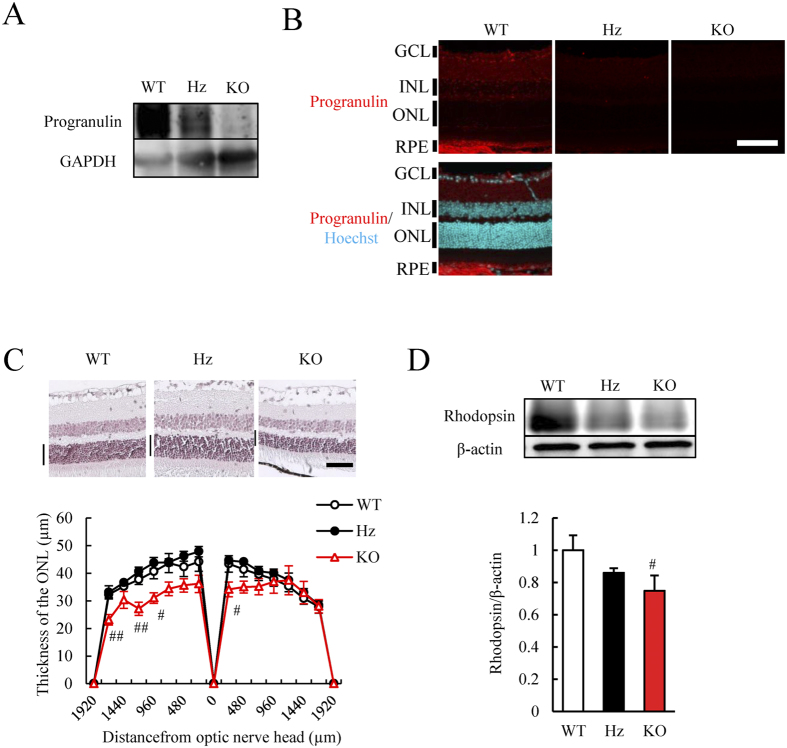
PGRN loss causes the developmental disorder in the ONL. (**A,B**) No expression of progranulin in the retina was observed in PGRN knockout (*Grn*^−/−^) mice. (**C**) ONL thickness was decreased in *Grn*^−/−^ mice compared to wild-type mice. Data are shown as means ± S.E.M. (n = 4 to 6). ^##^p < 0.01 and ^#^p < 0.05 vs. WT (Student’s *t*-test). (**D**) Rhodopsin expression was also decreased in Grn^−/−^ mice. Data are shown as means ± S.E.M. (*n* = 5). ^#^p < 0.05 vs. WT (Student’s *t*-test). WT: Wild-type; Hz: Heterozygous; KO: Knockout. Scale bar = 50 μm.

We measured retinal layer thickness in 8–12 week old PGRN-knockout mice^30^. We first confirmed that PGRN protein was not observed in *Grn*^−/−^ mice using western blotting and immunohistochemistry (Fig. 5A,B). The ONL thickness was significantly decreased in *Grn*^−/−^ mice compared to wild-type (WT) mice (Fig. 5C). The ONL thickness of *Grn*^−/−^ mice at 4 weeks old also tended to be decreased compared to heterozygous PGRN-knockout (*Grn*^+/−^) mice at the same age (Supplementary Fig. S6A–C). The cell number in the GCL was also decreased in *Grn*^−/−^ mice (Supplementary Fig. S8A,B). There were no significant changes in any of the retinal layers in *Grn*^+/−^ mice (Fig. 5C and Supplementary Fig. S8). In this period, the cell proliferation was not occurred because of Ki-67 negative in all retinal cell layer of *Grn*^−/−^ and WT mice (Supplementary Fig. S7). Western blotting demonstrated a decrease in rhodopsin expression in *Grn*^−/−^ mice compared to WT mice (Fig. 5D). These results suggest that the retina in *Grn*^−/−^ mice exhibited abnormal photoreceptor cell development.

## Discussion

It has been reported that adult mammals slightly show a limited potential for regeneration of retinal neurons after injury^19^. Brain-derived neurotrophic factor (BDNF) treatment following MNU-induced retinal damage has been shown to promote Müller glial cell proliferation and differentiation to photoreceptor cells^17^. In the present study, we also used an MNU-induced retinal injury model to investigate whether ASC-CM, containing PGRN and the other factors promotes the proliferation of retinal precursor cells and the differentiation to photoreceptor cells because BrdU^+^ cells are increased by the treatment of ASC-CM and PGRN and furthermore BrdU^+^ cells were colocalized with photoreceptor marker or retinal precursor cell marker. These findings suggest that PGRN and ASC-CM were associated with the migration and the proliferation of or the differentiation of retinal photoreceptor precursor cells after retinal damage in mice. In a previous report, we showed that ASC-CM contains a number of growth factors (HGF and activin A etc.) in addition to PGRN^15^. Activin A promotes the differentiation of photoreceptor cells *in vitro*^31^. The deletion of the HGFR gene impairs liver regeneration through a decrease in oval cell migration and hepatocytic differentiation^32^. HGF is also associated with axonal regeneration after optic nerve crush^33^. On the basis of these results and those from previous reports, it appears that ASC-CM containing PGRN, HGF, and activin A may exert multiple effects on retinal precursor cells and promote their differentiation to rhodopsin^+^ photoreceptor cells following retinal damage (Fig. 1B–D). However, PGRN treatment alone did not result in the full regeneration of photoreceptor cells *in vivo* (Fig. 2 and Supplementary Figs S2–4). PGRN increased BrdU^+^ cells in the ONL and the very few of these were Rx^+^ retinal precursor cells (Fig. 2). An HGFR inhibitor suppressed the differentiation to photoreceptor cells promoted by PGRN (Fig. 4D–G). Previous reports have shown that PGRN treatment can induce the phosphorylation of HGFR in cultured cell line^15^, which is consistent with the PGRN induced phosphorylation of HGFR found in the present study (Fig. 4B). Zebrafish *GrnA* (an orthologue of mammalian PGRN) knockdown decreased the protein expression of HGFR and downstream β-catenin^15^,^21^,^34^, suggesting that PGRN is closely involved in HGFR signaling. HGFR is associated with oval cell migration^32^ and the proliferation and migration of myogenic precursor cells^35^. The activation of the HGFR pathway by PGRN may result in the proliferation and the migration of Rx^+^ retinal precursor cells into the ONL.

PGRN promoted differentiation to rhodopsin^+^ photoreceptor cells and resulted in a decrease in CRX^+^ photoreceptor precursor cells and DCX^+^ neural precursor cells (Fig. 3C–F). Some reports have shown that PGRN may be involved in hepatocyte growth factor receptor (HGFR) and Wnt/β-catenin signaling^10^,^15^,^34^, and an association between HGFR and Wnt signaling has been suggested^36^,^37^. The activation of the Wnt signaling pathway promotes Müller glial cell proliferation and dedifferentiation^14^, whilst inhibition of Wnt signaling promotes neuronal differentiation^38^. On the other hand, Wnt activation increases adult hippocampal neurogenesis by increasing DCX^+^ cells and Tuj1^+^ mature neurons^13^. However, the association between Wnt signaling and neuronal differentiation remains controversial.

The present study showed that the thickness of the ONL was also decreased in younger *Grn*^−/−^ mice retina (Fig. 5C,D). A previous report showed that the thickness of the ONL was decreased in 12 months old *Grn*^−/−^ mice^39^ with the accumulation of retinal lipopigments. The decrease in the ONL thickness in *Grn*^−/−^ mice suggests that endogenous PGRN is essential for retinal photoreceptor cell development. These results show that PGRN may play a key role in photoreceptor cell development. However, PGRN may also be associated with the survival of retinal precursor cell or retinal precursor cell proliferation and migration considering from the result of PGRN treatment after retinal damage or in primary retinal cells in present study (Figs 2 and 3). PGRN deletion may decrease the ONL thickness through a broader effect. Further investigation should clarify whether PGRN is associated with photoreceptor cell development.

**Figure 6:**
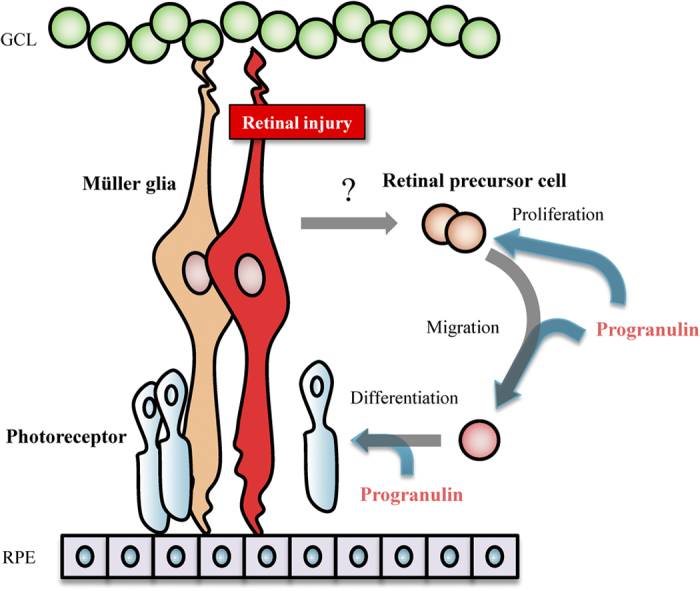
A putative association between PGRN and retinal regeneration. Retinal injury induces the dedifferentiation of Müller glia. Müller glial reprogramming generates retinal precursor cells. Generated retinal precursor cells have the potential to migrate to any retinal layer and differentiate into various retinal cells. PGRN can promote the migration of retinal precursor cells to the ONL and encourage their differentiation to photoreceptor cells.

Previous reports have shown that the combination of Wnt and retinoic acid or valproic acid promote differentiation to photoreceptor cells^14^. Valproic acid treatment after lentivirus-mediated expression of Sox2 (sex determining region Y-box 2) promotes the maturation of neurons in the brain^18^. The present findings demonstrate that ASC-CM and PGRN may be associated with the migration and the proliferation of retinal precursor cells and their differentiation to photoreceptor cells (Fig. 6). Retinal regeneration proceeds through two pathways, namely dedifferentiation (of Müller glia) and differentiation (of retinal precursor cell). The combination of factors promoting dedifferentiation and PGRN may encourage the regeneration of photoreceptor cells after injury.

## Materials and Methods

### Animals

Male adult C57BL/6, ddY mice, female ddY pregnant mice, and neonatal mice (Japan SLC, Hamamatsu, Japan) were maintained under controlled lighting environment (12 h/12 h light/dark cycle). PGRN knockout mice generated by the technique of Kayasuga *et al.*^30^ were obtained from Riken BioResource Center (Tsukuba, Japan) and were backcrossed with C57BL/6 mice. Genotyping was performed as described in the Data Sheet provided by the Riken BioResource Center. All experiments were performed in accordance with the ARVO Statement for the Use of Animals in Ophthalmic and Vision Research and were approved and monitored by the Institutional Animal Care and Use Committee of Gifu Pharmaceutical University.

### ASC isolation, culture and the collection of CM

Murine ASCs were obtained from a C57BL/6-Tg (CAG-EGFP) mouse that ubiquitously expresses enhanced green fluorescent protein (EGFP) as previously reported^15^. Adipose tissue was taken from a subcutaneous site. The inguinal fat pads were removed for ASC culture and the tissue including ASC was obtained as previously described^40^. The fat tissue was cut up with a blade, digested with 0.15% collagenase (Wako Pure Chemical Industries, Ltd., Osaka, Japan), and centrifuged. The cell pellet was re-suspended in 10% fetal bovine serum (FBS: Thermo Scientific, Waltham, MA, USA)/Dulbecco’s modified Eagle’s medium (DMEM: NacalaiTesque Inc, Kyoto, Japan) and plated onto a 100-mm culture dish. ASCs were maintained in 10% FBS/DMEM, 100 U/mL penicillin (Meiji Seika Pharma Co., Ltd., Tokyo, Japan), and 100 μg/mL streptomycin (Meiji Seika) in a humidified atmosphere of 95% air and 5% carbon dioxide (CO_2_) at 37 °C. The cells were passaged by trypsinization every 2–3 days with cells from passages 4 to 8 harvested for use in experiments. For the collection of CM, ASCs (4 × 10^5^ cells) were cultured in FBS-free DMEM. ASC-CM were collected after 72 h of culture, centrifuged at 300 × *g* for 5 min and filtered using a 0.22-μm syringe filter. The media were concentrated by centrifugation at 2,600 × *g* using the Amicon Ultra-15 units (Millipore, Bedford, MA, USA; molecular weight cutoff: 3,000).

### Primary retinal cell culture

Retinas from P8 ddY mice were dissected to remove the choroidal vessels and the cells were dissociated by incubating for 20 min in pre-activated papain at 37 °C according to the protocol used in our previous report^41^. Neurobasal medium (Invitrogen, Carlsbad, CA, USA), including ovomucoid (Sigma-Aldrich, St. Louis, MO, USA) and DNase (Sigma-Aldrich) was added to the cells. The cells were then centrifuged at 522 × *g* for 8 min at room temperature. The pellet was suspended in neurobasal medium including ovomucoid without DNase and re-centrifuged. The cells were then resuspended in neurobasal medium containing L-glutamine, B27 supplement (Invitrogen) and antibiotics. Cells were plated onto poly-D-lysine (sigma)/laminin (corning)–coated 12-well plate at a concentration of 2.0 × 10^6^ cells/well and onto glass chamber slides at 1.0 × 10^6^ cells/well. After incubation for 20 h, the medium was changed to neurobasal medium containing L-glutamine, B27 minus antioxidants (Invitrogen) and antibiotics. At 1 h prior to PGRN treatment, an HGFR inhibitor, SU11274 (Merck & Co., Whitehouse Station, NY, USA) was added at a final concentration of 1 μM. Recombinant mouse PGRN (R&D systems, minneapolis, MN, USA) was added at a final concentration of 500 ng/mL. The vehicle-treated group was treated medium alone. Three days after the cells had been isolated, the medium was changed with the same additions as outlined above. After 5 days the cells were harvested and used for western blotting or immunostaining.

### *N*-methyl-*N*-nitrosourea (MNU)-induced retinal damage model *in vivo*

Male ddY mice were injected 60 mg/kg MNU (Sigma-Aldrich) by intraperitoneal (i.p.) injection according to our previous procedure^41^. Mice were also injected 50 mg/kg 5-Bromo-2′-deoxyuridine (BrdU: Sigma-Aldrich) by i.p. injection. After this treatment, mice were injected 150-fold concentrated ASC-CM (2 μL) by intravitreal (i.v.) injection in the left eye under general anesthesia. Mice were anesthetized with 3.0% isoflurane (Merck Animal Health, Boxmeer, The Netherlands) and maintained with 1.5% isoflurane in 70% nitrous oxide and 30% oxygen by using an animal general anesthesia apparatus (Soft Lander; Sin-ei Industry Co., Ltd., Saitama, Japan). For the vehicle-treated (control) group, mice were injected with 150-fold concentrated DMEM (2 μL). At 2 and 4 days after MNU treatment, mice were treated with BrdU and ASC-CM or vehicle similarly. After 5 days, the eyes were enucleated and used for immunohistochemistry.

### Light-induced retinal damage model *in vivo*

We have previously performed the evaluation using a light-induced retinal degeneration model^15^,^42^,^43^. The mice underwent dark adaptations for 24 h, the pupils of the mice were then dilated using 1% cyclopentolate hydrochloride eye drops (Santen, Osaka, Japan) 30 min before exposure to light. Non-anesthetized mice were exposed to 8,000 lx of white fluorescent light (Toshiba, Tokyo, Japan) for 3 h in cages with a reflective interior. Following the light exposure, mice were injected 50 mg/kg BrdU (Sigma-Aldrich) by i.p. injection. After BrdU treatment, in the dark, mice were injected with recombinant mouse PGRN (R&D systems) 250 μg/mL (2 μL) by intravitreal (i.v.) injection in the left eye under general anesthesia. Mice were anesthetized as described above. In the vehicle-treated (control) group, mice were injected with D-PBS (Wako Pure Chemical Industries, Ltd.). The temperature during exposure to light was maintained at 25 °C ± 1.5 °C. The animals were kept in darkness for 24 h after light exposure. The mice were then returned to a normal light/dark cycle. At 2 and 4 days after light exposure mice were treated with BrdU and PGRN or vehicle. After 5 days eyes were enucleated and used for immunohistochemistry.

### *In vivo* immunostaining

The enucleated eyes were fixed in 4% paraformaldehyde for 24 h at 4 °C. The eyes were then incubated in 25% sucrose for 48 h at 4 °C and embedded in optimal cutting temperature compound (Sakura Finetechnical Co., Ltd., Tokyo, Japan). Eyes were cut in transverse cryostat sections of 10 μm thickness and placed on glass slides (MAS COAT; Matsunami Glass Ind., Ltd., Osaka, Japan). The retinal sections were blocked using non-immune serum [goat serum, horse serum (Vector Labs)] for 1 h and incubated with the primary antibody at 4 °C overnight. For the mouse antibody, M.O.M immunodetection kits (Vector Labs, Burlingame, CA, USA) were used for blocking and solvent. After being left overnight, the sections were incubated with a secondary antibody for 1 h. They were then counterstained for 5 min using Hoechst 33342 (1:2000 dilution: Invitrogen). Finally, they were mounted in Fluoromount (Diagnostic BioSystems, Pleasanton, CA, USA).

For BrdU staining, the retinal sections were pre-treated for 30 min with 2M hydrochloric acid (HCl) 2M for 30 min. They were incubated with 0.3% Triton X-100 (Bio-Rad Labs, Hercules, CA, USA) for 30 min. They were then treated with 0.1% trypsin (Wako Pure Chemical Industries, Ltd.) at 37 °C for 10 min.

The following antibodies were used: rat anti-BrdU [1:200 dilution: Abcam (Cambridge, MA, USA)], mouse anti-rhodopsin (1:1000 dilution: Millipore), mouse anti-GFAP [1:100 dilution: SantaCruz (Dallas, Texas, USA)], rabbit anti-Iba-1 (1:50 dilution: Wako Pure Chemical Industries, Ltd.), rabbit anti-RAX (Rx) (1:300 dilution: Abcam), mouse anti-Pax6 (1:500 dilution: Abcam), sheep anti-progranulin (1:20 dilution: R&D systems), Alexa Fluor^®^546 goat anti-rat IgG, Alexa Fluor^®^488 goat anti-mouse IgG, Alexa Fluor^®^633 goat anti-mouse IgG, Alexa Fluor^®^633 goat anti-rabbit IgG, and Alexa Fluor^®^647 donkey anti-sheep IgG (Invitrogen).

Images were acquired using a confocal microscope (FLUOVIEW FV10i; Olympus, Tokyo, Japan). For quantitative data images were taken 500 μm superior from the optic nerve. The total number of immunoreactive cells was counted within the entire area of the image (211.968 × 211.968 μm). The number was calculated as number/mm.

### *In situ* hybridization

Retinas from P5 mice were dissected and RNA extraction was performed using NucleoSpin® RNA II [Takara Bio Inc. (Shiga, Japan)]. Digoxigenin (DIG)-labelled antisense RNA riboprobe was prepared by *in vitro* transcription from pGEM®-T Easy Vector [Promega (Madison, WI, USA)] containing cDNA sequences of the mouse Rx. DIG RNA Labeling Kit (SP6/T7) [Roche Diagnostics (Basel, Switzerland)] was used for the making of RNA probes. RNA probes were hydrolyzed by carbonate buffer for 60 min and diluted in hybridization buffer. Retinal sections on slides were pretreated by proteinase K for 4 min. After washing slides, the sections were dried in air at least 1 hour. Sections were hybridized with probes in hybridization buffer overnight at 65  °C in a humidified box. Next, RNase was used for the elimination of unnecessary RNA. Then, blocking was performed with 1X Maleate/0.05% Triton/1 X Denhardt’s solution (Sigma-Aldrich) for 2 hours. Sections were incubated with the anti-DIG antibody (1:5000 dilution) (Roche Diagnostics) overnight at room temperature. After the incubation, NBT/BCIP solution (pH 9.5) was used for the color reaction. When color reaction was completed, BrdU and Rx staining was performed according to above method.

Mouse Rx primer sequence (5′-3′)

Forward: GCTTCTCGCTCGCTGGCCAC

Reverse: CTTCCAGCGAGAACTTGTCC

### *In vitro* immunostaining

The primary retinal cultures were fixed with 4% paraformaldehyde at room temperature for 15 min. The cells were then incubated with 0.2% Triton X-100 (Bio-Rad Labs) in PBS for 10 min and 50 mM glycine (Wako) in PBS for 15 min. The cells were blocked with 3% goat serum or horse serum (Vector Labs) for 30 min and incubated with the primary antibodies overnight at 4 °C. The cells were then incubated for 1 h with secondary antibodies and counterstained with Hoechst 33342 (Invitrogen). Finally, the cells were mounted in Fluoromount (Diagnostic BioSystems) and imges were taken using a confocal microscope (FLUOVIEW FV10i). For quantitative data, the images were obtained within the entire area of the image (211.968 × 211.968 μm). The number of immunoreactive cells was counted and calculated as the ratio of immunoreactive cells to total cells.

The following antibodies were used: mouse anti-rhodopsin (1:1000 dilution: Millipore), rabbit anti-CRX (1:20 dilution: SantaCruz), goat anti-DCX (1:20 dilution: SantaCruz), mouse anti-nestin [1:100 dilution: BD Bioscience (San Jose, CA, USA)], Alexa Fluor^®^488 goat anti-mouse IgG, Alexa Fluor^®^546 donkey anti-rabbit IgG, Alexa Fluor^®^488 donkey anti-goat IgG, and Alexa Fluor^®^633 goat anti-mouse IgG (Invitrogen).

### Western blot analysis

Primary retinal cells or mice retinas were lysed using a buffer (RIPA buffer; Sigma-Aldrich) containing protease (Sigma-Aldrich) and a phosphatase inhibitor cocktail (Sigma-Aldrich). To extract retinal protein, the tissue was homogenized in cell-lysis buffer using a Physcotron homogenizer (Microtec Co., Ltd., Chiba, Japan). The cell lysate was centrifuged at 12,000 × *g* for 20 min, and the supernatant was used for subsequent experiments. Protein concentration was measured by comparison with a known concentration of BSA using a bicinchoninic acid protein assay kit (Thermo Scientific). A mixture of equal parts of protein and sample buffer with 10% 2-mercaptoethanol (Wako Pure Chemical Industries, Ltd.) was subjected to SDS-PAGE using 5–20% gradient gels (Wako Pure Chemical Industries, Ltd.). The separated proteins were transferred onto a polyvinylidene difluoride membrane (Immobilon-P: Millipore Corporation, Billerica, MA, USA). After blocking for 30 min at room temperature with Block One-P (Nacalai Tesque, Inc., Kyoto, Japan), membranes were washed in 10 mM Tris-buffered saline containing 0.05% Tween 20 and then incubated with the primary antibody overnight at 4 °C. The following primary antibodies were used: mouse anti-rhodopsin (1:1000 dilution: Millipore), sheep anti-PGRN (1:100 dilution: R&D systems), and mouse anti-β-actin (1:2,000 dilution: Sigma-Aldrich). After exposure to the primary antibody, the membranes was incubated with peroxidase goat anti-rabbit, goat anti-mouse or rabbit anti-sheep IgG (Thermo Scientific) as the secondary antibody. The immunoreactive bands were visualized using an ImmunoStar LD (Wako Pure Chemical Industries, Ltd.).

### Histological analysis

PGRN-knockout and WT mice eyes were enucleated and fixed in 4% paraformaldehyde for 24 h at 4 °C. Six paraffin-embedded sections (5 μm thickness) cut through the optic disc of each eye were prepared in a standard manner and stained with hematoxylin and eosin. Six sections from each eye were used for the morphometric analysis. Light microscopy images were taken and the thickness of the ONL was measured at 240 μm intervals from the optic disc in a blind manner by H.I. The data was averaged for each eye.

### Statistical analysis

The data is presented as mean ± S.E.M. Statistical comparisons were conducted using a two-tailed Student’s *t*-test in all Figures except for Fig. 6d [STAT VIEW version 5.0 (SAS Institute, Cary, NC, USA)]. *p* < 0.05 was considered as statistically significant.

The number of samples (animals) indicates ‘n’ in each figure legend.

## Additional Information

**How to cite this article**: Kuse, Y. *et al.* Progranulin promotes the retinal precursor cell proliferation and the photoreceptor differentiation in the mouse retina. *Sci. Rep.* **6**, 23811; doi: 10.1038/srep23811 (2016).

## Supplementary Material

Supplementary Information
