# Supplementary material for: Progranulin promotes the retinal precursor cell proliferation and the photoreceptor differentiation in the mouse retina
Source: Sci Rep. 2016 Mar 31;6:23811. doi: 10.1038/srep23811 (PMC4814875; doi:10.1038/srep23811)
Supplement: Supplementary Information [file srep23811-s1.pdf]

Supplementary information

Progranulin promotes the retinal precursor cell proliferation and the photoreceptor differentiation in the mouse retina.

Yoshiki Kuse, Kazuhiro Tsuruma, Sou Sugitani, Hiroshi Izawa, Yuta Ohno, Masamitsu Shimazawa, Hideaki Hara*

Molecular Pharmacology, Department of Biofunctional Evaluation, Gifu Pharmaceutical University, 1-25-4 Daigaku-nishi, Gifu 501-1196, Japan.

*For reprints and all correspondence: Professor H. Hara, Ph.D., R.Ph., Molecular Pharmacology, Department of Biofunctional Evaluation, Gifu Pharmaceutical University, 1-25-4 Daigaku-nishi, Gifu 501-1196, Japan.

Supplementary Methods

***In vivo* immunostaining**

The enucleated eyes were fixed in 4% paraformaldehyde for 24 h at 4° C. The eyes were then incubated in 25% sucrose for 48 h at 4° C and embedded in optimal cutting temperature compound (Sakura Finetechnical Co., Ltd., Tokyo, Japan). Eyes were cut in transverse cryostat sections of 10 μm thickness and placed on glass slides (MAS COAT; Matsunami Glass Ind., Ltd., Osaka, Japan). The retinal sections were blocked using non-immune serum [goat serum, horse serum (Vector Labs)] for 1 h and incubated with the primary antibody at 4° C overnight. For the mouse antibody, M.O.M immunodetection kits (Vector Labs, Burlingame, CA, USA) were used for blocking and solvent. After being left overnight, the sections were incubated with a secondary antibody for 1 h. They were then counterstained for 5 min using Hoechst 33342 (1:2000 dilution: Invitrogen). Finally, they were mounted in Fluoromount (Diagnostic BioSystems, Pleasanton, CA, USA).

For BrdU staining, the retinal sections were pre-treated for 30 min with 2M hydrochloric acid (HCl) 2M for 30 min. They were incubated with 0.3% Triton X-100 (Bio-Rad Labs, Hercules, CA, USA) for 30 min. They were then treated with 0.1% trypsin (Wako Pure Chemical Industries, Ltd.) at 37° C for 10 min.

The following antibodies were used: rat anti-BrdU [1:200 dilution: Abcam (Cambridge, MA, USA)], goat anti-Brn3a [1:100 dilution: SantaCruz (Dallas, Texas, USA)], mouse anti-GFAP (1:100 dilution: SantaCruz), mouse anti-nestin [1:100 dilution: BD Bioscience (San Jose, CA, USA)], rabbit anti-Sox2 antibody [1:100 dilution: Millipore (Billerica, MA, USA)], rabbit anti-CRX (1:20 dilution: SantaCruz), rabbit anti-Ki-67 antibody (1:200 dilution: Millipore), Alexa Fluor®488 goat anti-rabbit IgG, Alexa Fluor®546 goat anti-rat IgG, Alexa Fluor®488 donkey anti-goat IgG, Alexa Fluor®488 goat anti-mouse IgG, Alexa Fluor®633 goat anti-mouse IgG, Alexa Fluor®633 goat anti-rabbit IgG (invitrogen). Images were acquired using a confocal microscope (FLUOVIEW FV10i; Olympus, Tokyo, Japan). For quantitative data images were taken 500 μm superior from the optic nerve. The total number of immunoreactive cells was counted within the entire area of the image (211.968 × 211.968 μm). The number was calculated as number/mm.

Histological analysis

PGRN-knockout and WT mice eyes were enucleated and fixed in 4% paraformaldehyde for 24 h at 4° C. Six paraffin-embedded sections (5 μm thickness) cut through the optic disc of each eye were prepared in a standard manner and stained with hematoxylin and eosin. Six sections from each eye were used for the morphometric analysis. Light microscopy images were taken and the cell number of GCL or thickness of the IPL, INL, and OPL was measured at 500 μm from the optic disc in a blind manner by H.I.. The data was averaged for each eye.

Supplementary Figure S1

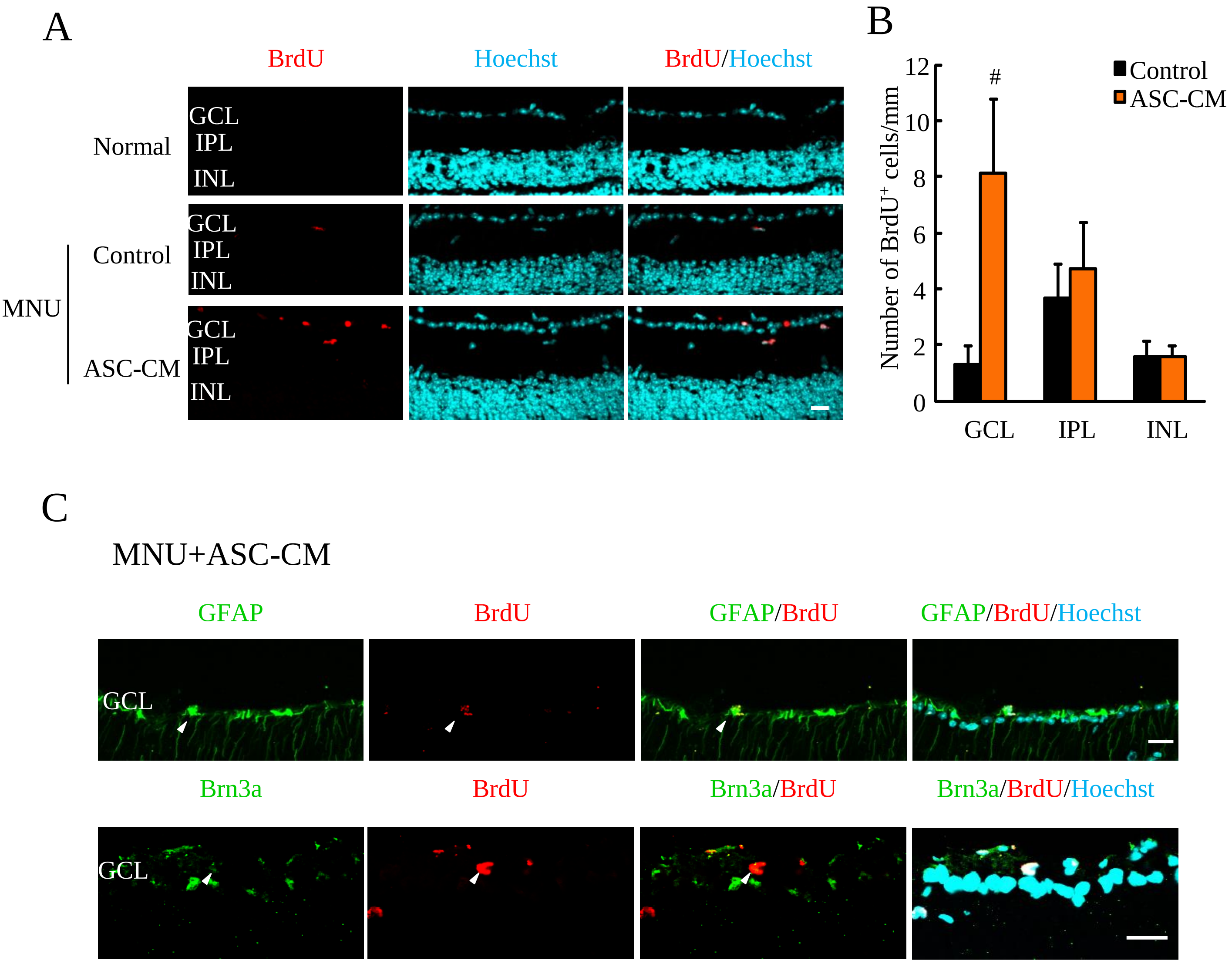

Supplementary Figure S1 The effect of ASC-CM on the retinal layer excluding ONL. (A) Typical images show BrdU (red) and Hoechst 33342 (cyan) staining. There are large numbers of $BrdU^+$ cells in the GCL in the ASC-CM-treated group. No change to the IPL and or INL was observed. (B) Quantitative data showing that ASC-CM addition increased the number of $BrdU^+$ cells in the GCL compared to control group. (C) $BrdU^+$ cells increased by ASC-CM in GCL are colocalized to GFAP (green) but not Brn3a (green). Data are shown as means ± S.E.M. (n = 6). $^{\#}$ $p < 0.05$ vs. Control (Student's *t*-test). Scale bar = 20 μm.

Supplementary Figure S2

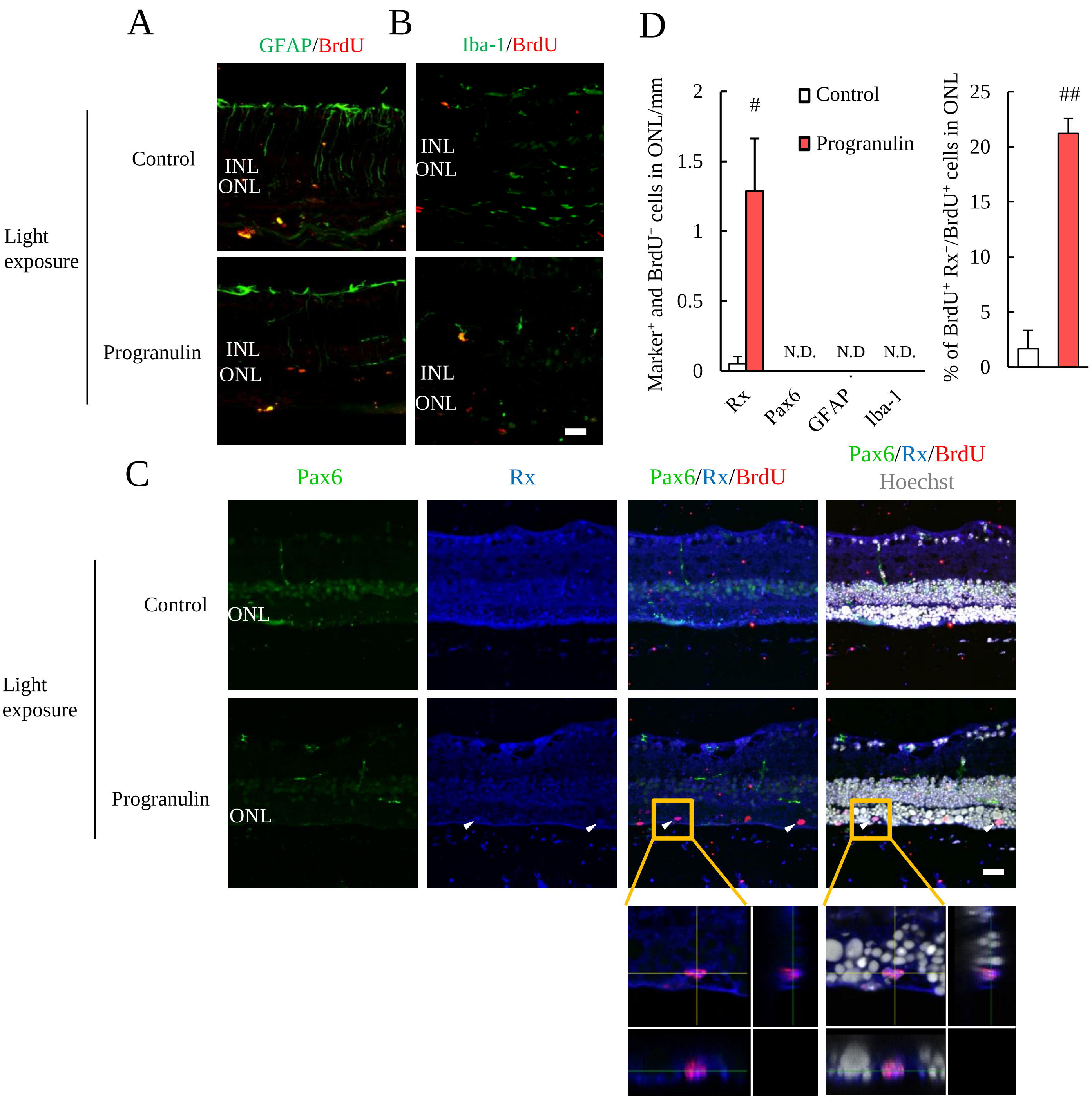

Supplementary Figure S2 PGRN treatment increased Rx⁺ precursor cells in the ONL.
(A) GFAP (an astrocyte marker) expression (green) was observed in the retinal inner layer and no BrdU (red) staining of astrocytes was evident in the ONL in either the PGRN-treated group or the control group. (B) Iba-1 (a microglia marker) expression (green) in the ONL did not stain BrdU positive in the ONL in either the PGRN-treated group or the control group. (C) Pax6 (green) and Rx (blue) are retinal precursor cell markers. Pax6 staining was absent in the ONL. In PGRN-treated group, some $BrdU^+$ cells were Rx positive in the ONL. $BrdU^+$ Rx^+ cells were confirmed by confocal imaging. (D) Quantitative data shows the presence of $BrdU^+$ Rx^+ cells in the ONL as a result of PGRN treatment and the absence of $BrdU^+$ Rx^+ cell in the control group Data are shown as means ± S.E.M. (n = 5 or 6). $^{\#\#}$; $p < 0.01$ and $^{\#}$; $p < 0.05$ vs. control (Student's *t*-test). Scale bar = 20 μm

Supplementary Figure S3

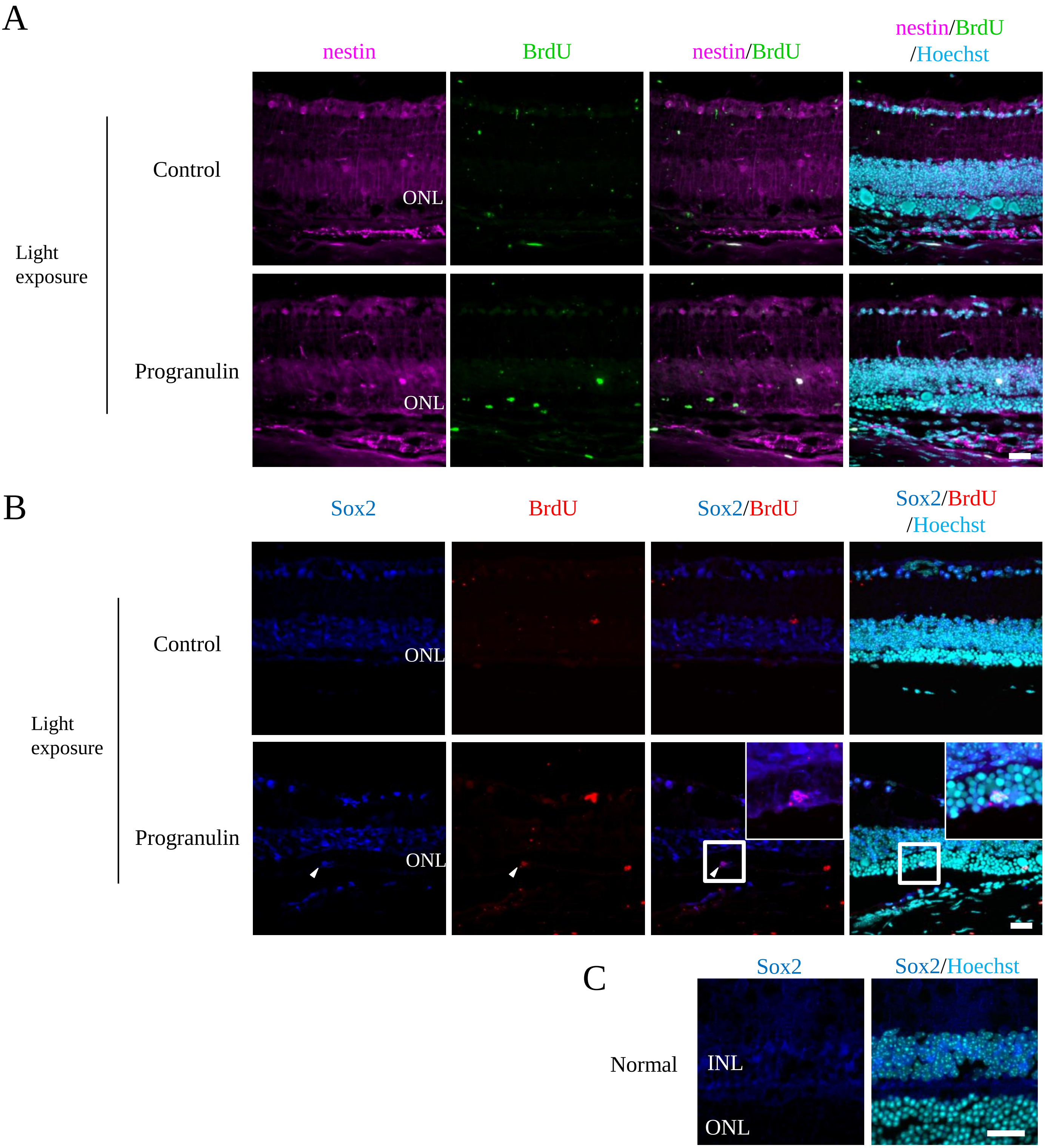

Supplementary Figure S3 PGRN treatment after light damage did not alter the expression of nestin, but generated the BrdU$^+$Sox2$^+$ cells in ONL.

(A) Light-induced retinal damage activated the nestin (magenta) expression and progranulin treatment did not alter the expression. (B) Sox2 (blue) expression was not completely observed in vehicle-treated group. Progranulin induced the newly-generated Sox2$^+$ cell. (C) Sox2 expression in normal retina. Data are shown as means ± S.E.M. (n = 3). Scale bar = 20 μm.

Supplementary Figure S4

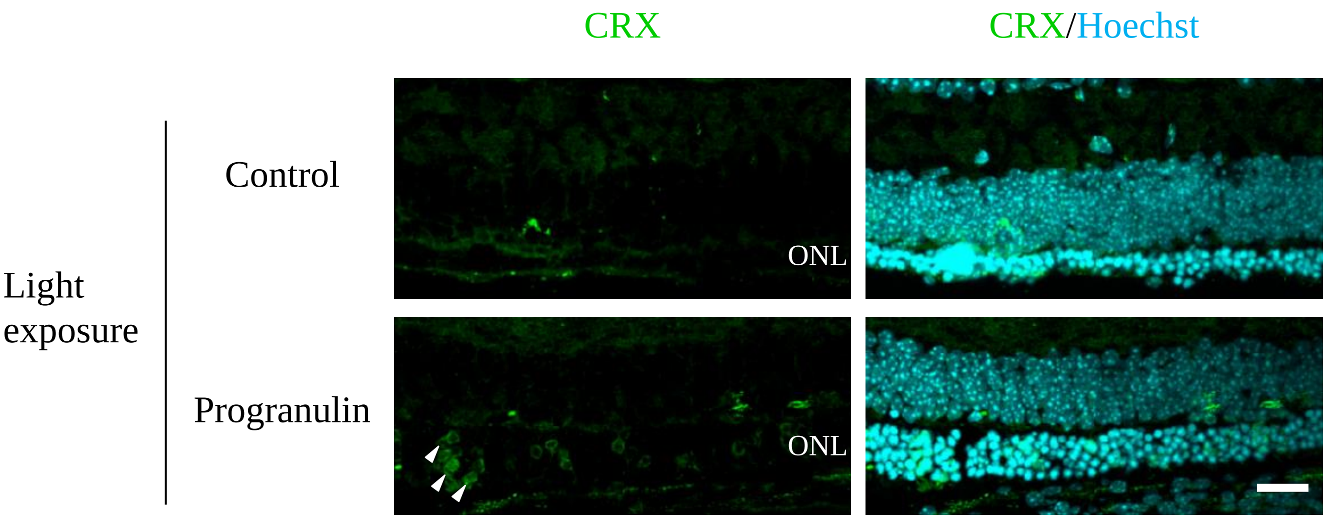

Supplementary Figure S4 PGRN treatment after light damage increased the CRX$^+$ cells in ONL.
CRX (green) expression was not completely observed in vehicle-treated group. Progranulin increased CRX$^+$ cell. Data are shown as means ± S.E.M. Scale bar = 20 μm.

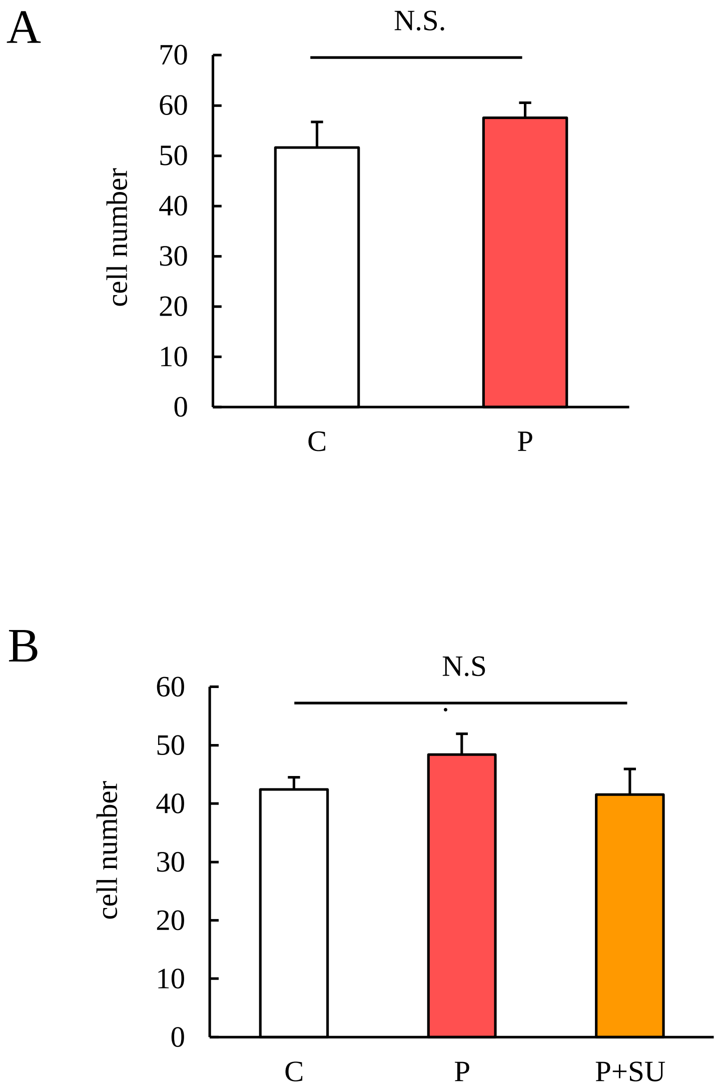

Supplementary Figure S5 The effect of PGRN to the cell number in retinal primary cell culture.

(A) The cell number (Hoechst positive cell number) in PGRN-treated group did not change compared to control. (B) The cell number in PGRN-treated group and PGRN and SU11274 co-treated group did not change compared to control. Data are shown as means ± S.E.M. (n = 3 or 4). Student's *t*-test.

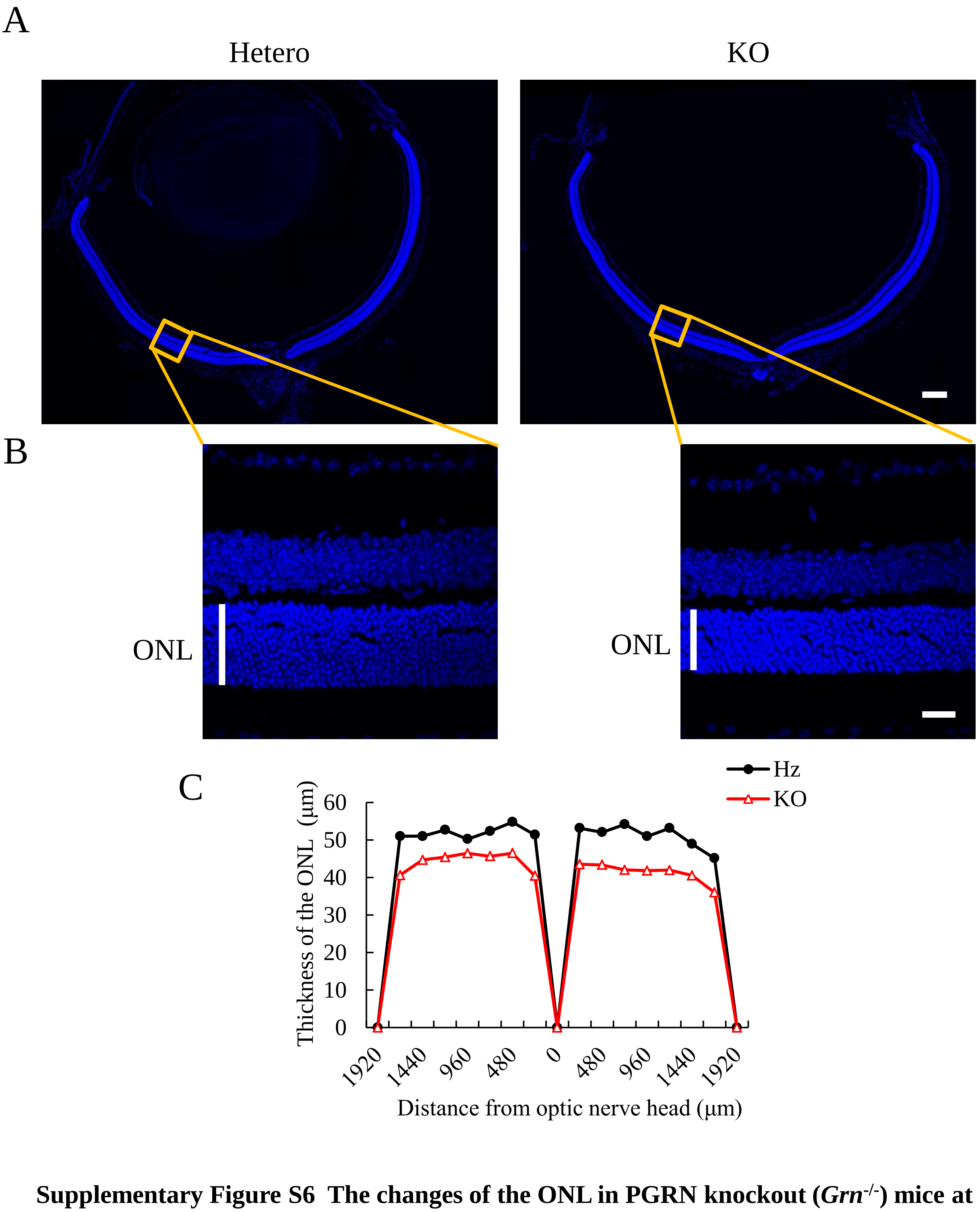

Supplementary Figure S6 The changes of the ONL in PGRN knockout (*Grn*$^{-/-}$) mice at 4 weeks old compared to heterozygous PGRN-knockout (*Grn*$^{+/-}$) mice.
(A) The whole retina stained by Hoechst. Scale bar = 200 µm. (B) The close-up image of retina. Scale bar = 20 µm. (C) The quantitative data of ONL thickness in PGRN knockout (*Grn*$^{-/-}$) mice and heterozygous PGRN-knockout (*Grn*$^{+/-}$) mice (Hetero n = 1, KO n = 2).

A

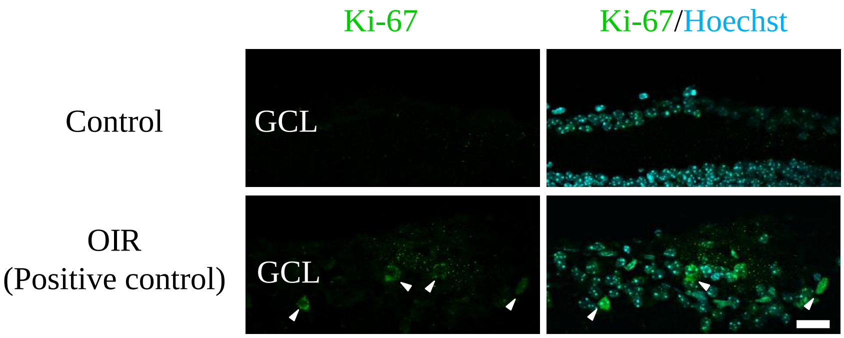

B

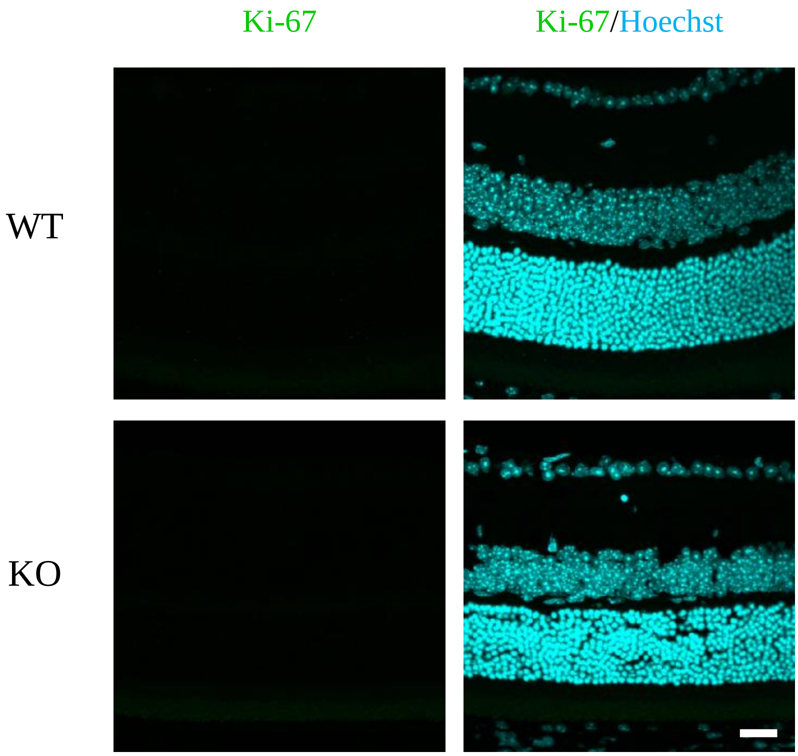

Supplementary Figure S7 The changes of Ki-67 expression in PGRN knockout (*Grn*$^{-/-}$) mice and wild-type mice.
(A) The immunostaining of Ki-67 using mice retina of oxygen-induced retinopathy (OIR) as the positive control. The retina in OIR group was observed Ki-67 positive cells but not control group. (B) The retina stained by Ki-67 (green) and Hoechst (cyan). No expression of Ki67 was observed in any retinal layer. Scale bar = 20 μm.

Supplementary Figure S8

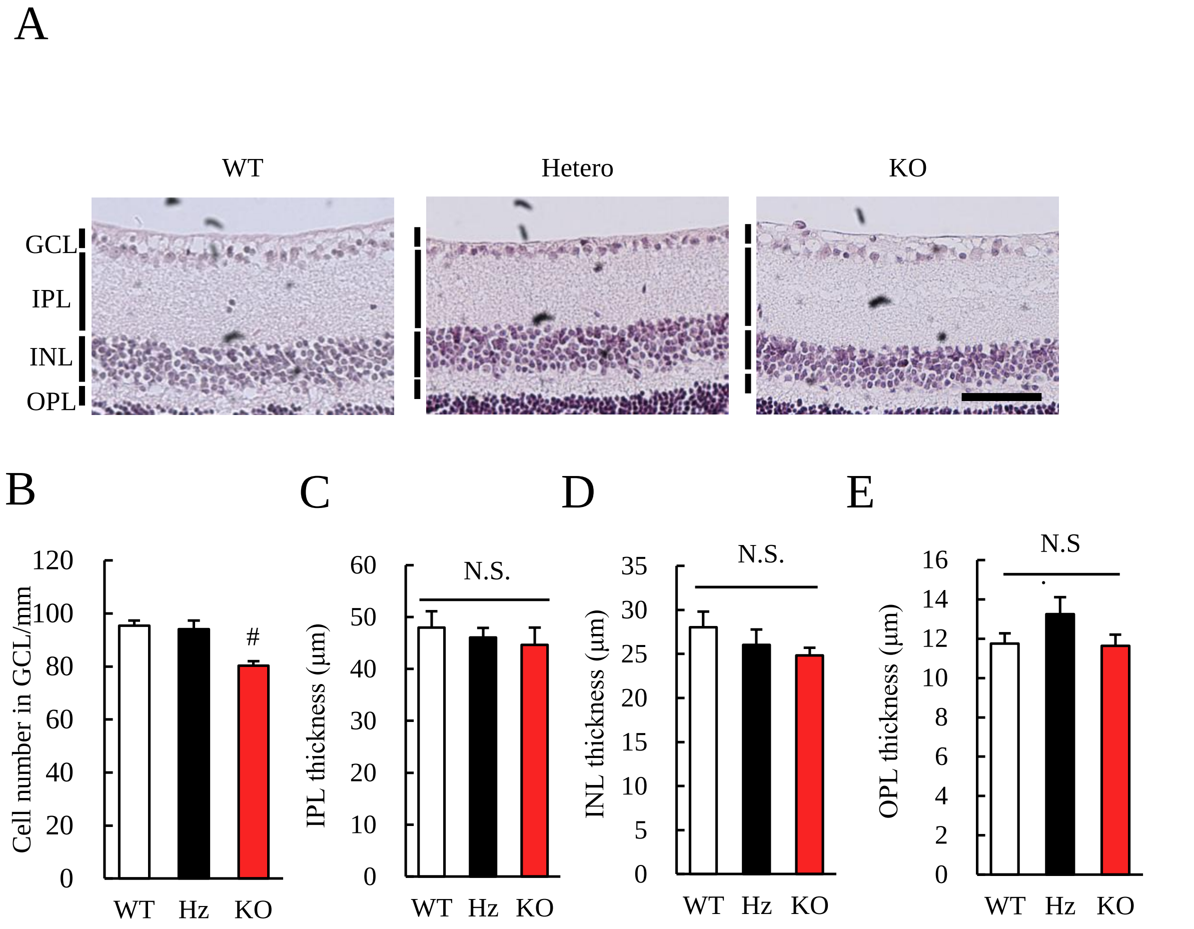

Supplementary Figure S8 The changes in the retinal layer excluding the ONL in PGRN knockout (*Grn*$^{-/-}$) mice.
(A) Typical images show the retinal inner layer in WT, Hz, and KO mice. (B-E) Quantitative data demonstrating the reduction in cell number in the GCL in KO mice compared to WT mice. There is no change in the IPL, INL or OPL. Data are shown as means ± S.E.M. (n = 5 to 9). $^{\#}$ $p < 0.05$ vs. WT (Student's *t*-test). Scale bar = 50 μm
